# Post‐traumatic stress disorder and beyond: an overview of rodent stress models

**DOI:** 10.1111/jcmm.13161

**Published:** 2017-04-04

**Authors:** Johanna Schöner, Andreas Heinz, Matthias Endres, Karen Gertz, Golo Kronenberg

**Affiliations:** ^1^ Klinik für Psychiatrie und Psychotherapie Charité ‐ Universitätsmedizin Berlin, Campus Charité Mitte Berlin Germany; ^2^ Center for Stroke Research Berlin (CSB) Charité ‐ Universitätsmedizin Berlin Berlin Germany; ^3^ Klinik und Hochschulambulanz für Neurologie Charité ‐ Universitätsmedizin Berlin Berlin Germany; ^4^ Klinik und Poliklinik für Psychiatrie und Psychotherapie Zentrum für Nervenheilkunde, Universitätsmedizin Rostock Rostock Germany

**Keywords:** animal model, FKBP5, psychosocial stress, PTSD, rodent

## Abstract

Post‐traumatic stress disorder (PTSD) is a psychiatric disorder of high prevalence and major socioeconomic impact. Patients suffering from PTSD typically present intrusion and avoidance symptoms and alterations in arousal, mood and cognition that last for more than 1 month. Animal models are an indispensable tool to investigate underlying pathophysiological pathways and, in particular, the complex interplay of neuroendocrine, genetic and environmental factors that may be responsible for PTSD induction. Since the 1960s, numerous stress paradigms in rodents have been developed, based largely on Seligman's seminal formulation of ‘learned helplessness’ in canines. Rodent stress models make use of physiological or psychological stressors such as foot shock, underwater trauma, social defeat, early life stress or predator‐based stress. Apart from the brief exposure to an acute stressor, chronic stress models combining a succession of different stressors for a period of several weeks have also been developed. Chronic stress models in rats and mice may elicit characteristic PTSD‐like symptoms alongside, more broadly, depressive‐like behaviours. In this review, the major existing rodent models of PTSD are reviewed in terms of validity, advantages and limitations; moreover, significant results and implications for future research—such as the role of FKBP5, a mediator of the glucocorticoid stress response and promising target for therapeutic interventions—are discussed.

PTSD is a disorder that may develop after exposure to a terrifying or catastrophic event involving actual or threatened death, serious injury or sexual violation. Typically, patients suffering from PTSD present symptoms of avoidance, intrusion and alterations in arousal, mood and cognition. Lifetime prevalence among adult Americans is 8.3% 1, being twice as high in women (11.0%) than in men (5.4%). In 1980, the diagnosis of PTSD was added to DSM‐3 2, as the severe psychological consequences following combat exposure became increasingly apparent, for example, in returning Vietnam Veterans 3.

PTSD is often accompanied by psychiatric co‐morbidities such as depression, substance abuse or anxiety disorders, which leads to a high level of functional disability. In addition, PTSD is a major risk factor for somatic diseases such as coronary heart disease 4 and stroke 5. It imposes an enormous economic burden on health services and society: In terms of medical care, loss of productivity and suicide, the financial impact of PTSD and major depression in American troops has been estimated to range from $4 to $6.2 billion over a 2‐year period post‐deployment 6.

Although a substantial body of research has greatly improved our knowledge regarding prevalence, clinical symptoms and consequences of PTSD over the last decades, relatively little is known about underlying neurobiological abnormalities. This is why various animal models have been proposed.

## General conditions for animal models in PTSD research

Willner 7 proposed three criteria to be considered regarding animal models: They should have a phenomenological resemblance to the modelled condition (‘face validity’), show the same underlying mechanisms as the human disorder (‘construct validity’) and make correct predictions about treatment effects (‘predictive validity’). Accordingly, as regard PTSD, the paradigms should include appropriate stressors which are able to provoke long‐lasting behavioural symptoms similar to PTSD, for example, hyperarousal, hypervigilance, social withdrawal and cognitive alterations (face validity). Furthermore, these symptoms should be ameliorated by established psychopharmacological treatments, for example selective serotonin reuptake inhibitors (SSRI), in order to meet the criterion of predictive validity. The biological pathomechanisms of PTSD are still far from being understood, so it is difficult to define exact conditions of construct validity. However, emerging evidence indicates that a number of biological factors that have been implicated in the development of PTSD in humans, for example altered inflammatory signalling or increased amygdala reactivity, should also be looked for in a potential animal model.

As depression and PTSD share a variety of etiological factors, it is not an easy task to develop an animal model that exclusively reflects the one but not the other disorder. First, there is a considerable overlap of symptoms, especially as regards the symptom cluster of ‘negative cognitions’: for example, the inability to experience positive emotions, numbing or alterations of sleep and concentration. Moreover, co‐morbidity between depression and PTSD is high 3, which, in practice, may make it quite difficult to make a clear distinction between the two disorders. Furthermore, it has been shown that psychosocial stress and adverse childhood events are not only risk factors for PTSD, but are also important contributors to the development of depressive disorders 8. The mere presence of a stressor in an experimental paradigm is thus not necessarily sufficient to reproduce characteristic PTSD behaviours, as it might just as well lead to depressive‐like symptoms. Yehuda and Antelman 9 proposed that the induction of a PTSD‐like syndrome should include a brief and very intense stressor, in contrast to more chronic and mild stressors in animal models of depression.

There may also exist some important differences between the biological underpinnings of depression and PTSD: While both disorders have been linked with dysregulation of the hypothalamic–pituitary–adrenal (HPA) axis, patients with depression typically display an increased level of plasma cortisol 10, whereas PTSD is associated with significantly lower concentration of plasma and urinary cortisol 11, 12. Therefore, it is hypothesized that PTSD leads to enhanced negative glucocorticoid feedback and hypocortisolism, a finding that may be highly specific for PTSD and, consequently, of major utility in the critical evaluation of experimental paradigms.

## Animal stress models: differences between rats and mice

Rodents have been particularly useful in experimental modelling of human disease. In 2014, the great majority of the experimental procedures carried out in the United Kingdom involved mice (76%) and rats (7%) 13. Rodents provide several advantages that make them convenient for researchers, for example easy housing and handling, a marked genetic resemblance to humans, fast reproduction and short lifespan. Although rats (Rattus Norvegicus) and mice (Mus Musculus) evolved from a common ancestor that lived 12–24 million years ago, they belong to different species that display numerous differences in anatomy, morphology, genetics and behaviour. Of special note, rat brains weigh approximately five times as much as mouse brains 14.

While rats have a clearly defined hierarchy system which finds expression through submissive posture or attack, mice do not typically present hierarchical behaviours 15. This point gains added significance in animal stress models that rely on social defeat (SD) or unstable housing conditions 16. In addition, rats and mice also seem to differ in how they cope with stress: Several studies have shown that stress models involving mice require comparably harsher stressors to reliably evoke anxiety‐like or depression‐like behaviours than stress models in rats 17. In summary, it is of major importance to keep species‐specific responses to stressors in mind when conceptualizing animal models of PTSD. Likewise, strain differences may play a crucial role in shaping behaviour and physiology and, therefore, also need to be taken into consideration.

## History: learned helplessness

In the late 1960s, Seligman and Maier 18 developed the so‐called ‘learned helplessness’ paradigm (LH), which is based on Pavlov's ‘classical conditioning’. They demonstrated that dogs which had previously been given inescapable electric shocks failed to escape in a different setting afterwards. Instead of jumping over a low fence in order to avoid further shocks, pre‐shocked dogs remained passive on the floor, which can be interpreted as a correlate of ‘behavioural despair’. The decreased locomotion and the lack of response initiative of the pre‐shocked animals can be conceptualized as a motivational deficit 19, which represents a core symptom of depressive disorders. This is why the LH procedure was primarily developed as a model of depression. Notwithstanding, different aspects of LH—namely, avoidance behaviour, numbing and the presence of a strong and inescapable stressor—were also recognized as closely linked with PTSD.

The concept of LH soon was extended to other species, especially rats and mice 20, 21. Van der Kolk reintroduced LH as an ‘Inescapable Shock‐Learned Helplessness’ (IS‐LH) paradigm of PTSD 22. Mice subjected to IS showed a relevant depletion of catecholamines and—putatively owing to changes in neurotransmitter concentrations—deficits in active motor responding performance 23. Moreover, it was shown that some psychopharmacological agents are able to reverse the long‐term sequelae of IS 24. IS‐LH has given rise to a controversial debate in the 1980s and early 1990s. Yehuda and Antelman 9, in particular, have called into question the usefulness of the paradigm since it fails to induce persisting behavioural changes, whereas long‐term duration and chronicity of PTSD constitute characteristic features in humans 25 and represent a key aspect of the diagnosis according to DSM‐5 26. This is why numerous modified models have emerged in the past decades, each offering a different approach to specific PTSD symptoms.

## Time‐dependent sensitization (TDS)

In 1988, Antelman described the TDS paradigm 27, which includes a very brief and intense stimulus, followed by a long‐lasting and progressively amplified response to subsequent pharmacological and non‐pharmacological stressors. Different types of stressor have been used in this model, mostly in rats: psychological stressors, for example, exposure to a black box 28 or immobilization 29, 30, as well as pharmacological stressors such as the injection of cocaine 29. The common feature of all these experiments is sensitization: A dangerous, potentially life‐threatening stimulus intensifies subsequent reactions to a similar or weaker stimulus, even if the second stimulus is applied much later 27. Under natural circumstances, sensitization is a fundamental reaction to threat as it accelerates escape responses and thus may protect the subject from future danger. The effects of TDS may be observed at multiple levels including long‐term changes in behaviour, neurotransmitter concentrations 31, neuroendocrine and immune‐system responses 30 as well as altered susceptibility to pharmacological interventions 31, 32.

## Foot shock (FS)

FS is a stressor broadly employed in animal models of anxiety, stress 21, 33, 34, 35, 36 and depression 18. FS elicits long‐lasting behavioural and physiological effects that persist up to 3 weeks and may even intensify over time 21. In general, animals are placed into a shock chamber on a metal‐grid floor and subjected to a sequence of electric shocks of variable intensity and duration. The FS model reproduces some of the core symptoms of PTSD, including avoidance and anxiety behaviour 33, hyperarousal, aggression and re‐experiencing 36, as well as alterations of sleep architecture 37. Some studies combine the FS model with a situational reminder (SR) of the stressful experience which serves as contextual conditioned Pavlovian cue (*e.g*. re‐exposure to the box where the shocks were administered). It has been shown that animals exposed to SR exhibit a distinct behavioural response (*e.g*. crouching near the back wall of the box, increase in respiratory rate, etc.) despite the absence of the stressor itself 36. The incorporation of SR in rodent models provides an interesting new research perspective as it recalls human PTSD symptoms such as re‐experiencing and intrusions.

## Underwater trauma (UT)

Richter‐Levin 38 developed a model of PTSD using UT. In this model, rats are forced to swim for 1 min. in a Morris water maze 39, from which the platform has been removed. Afterwards, they are held under water for 30 sec. with a metal net and then returned to a resting cage. Richter‐Levin argues that this kind of stressor is more ethologically relevant than electric shocks since the threat of drowning is quite real in the life of a rat living in the wild. Learning in the Morris water maze is impaired as late as 3 weeks after UT 38, a finding which is in line with various studies reporting negative effects of stress on memory retrieval and learning 40, 41. It also corresponds well to the ‘negative alterations in cognition’ criterion in DSM‐5 26.

## Single prolonged stress (SPS) and restraint stress (RS)

In the SPS paradigm 42, rats are subjected to RS for 2 hrs, which is followed immediately by a forced swim for 20 min., and, after a brief rest period, by exposure to ether vapours until the experimental animal loses consciousness. Besides the conventional immobilization on wooden boards, RS can also be achieved through a special restraining syringe 43. After a period of 7 days, rats are re‐stressed in a brief session of RS. SPS is the first experimental paradigm that was able to reproduce PTSD‐like alterations of the HPA axis 42. In rats previously exposed to SPS, an increased sensitivity to glucocorticoid fast feedback was demonstrated after re‐stress, a finding that recapitulates the HPA axis dysregulation commonly associated with PTSD: PTSD patients display significantly reduced urinary and salivary cortisol concentrations in tandem with increased suppression of cortisol in response to dexamethasone 11, 12. In contrast to increased sensitivity to glucocorticoid feedback in the SPS paradigm, a chronic stress model in rats yielded decreased feedback 44, which accords well with neuroendocrine findings reported in major depression 45, 46, 47. The stress–re‐stress model thus appears to be particularly suitable to compare the specific neuroendocrine pathomechanisms of PTSD *versus* depression.

In addition to its effects on HPA axis regulation, SPS has also been shown to influence neurotransmitter signalling. In particular, SPS reduces hippocampal NMDA receptor density and leads to a dysregulation of inhibitory GABA pathways 48, which may contribute to impairments in spatial memory. At the behavioural level, SPS leads to diminished fear extinction 49, anxiety‐like behaviour in the elevated plus maze and increased stress‐induced nociceptive sensitivity 50, symptoms that seem to be reversed by administration of SSRI 51, 52.

## Predator‐based stress

The confrontation with a natural predator, for example, cats, ferrets, rats or foxes, has been shown to provoke high levels of intense fear and stress in rats and mice, followed by long‐lasting behavioural and endocrine responses 53, 54. Predators and predator‐related stimuli such as the scent of predator urine are therefore highly effective tools for trauma induction in animal models. Typically, the rodent is exposed for a very brief period of time (5–10 min.) to the predator 16, 55 or to predator odorants, for example, soiled cat litter 56. A 5‐min. exposure of a rat to a cat leads to an increase in anxiety‐like behaviour in the rat in the elevated plus maze that is still detectable after 3 weeks 55. Zoladz and co‐workers modified the predator‐based stress model and devised the so‐called ‘predator‐based psychosocial stress paradigm’ (PPS) 16, which combines acute predator exposure with unstable housing conditions (*i.e*. randomly changing cage partners). The authors hypothesize that the chronic stress associated with unstable housing conditions exacerbates the psychological sequelae of predator exposure in much the same way that the likelihood of developing chronic PTSD is increased in trauma victims that lack social support 57. Similar to the changes induced by SPS, PTSD‐like glucocorticoid abnormalities with reduced corticosterone levels at baseline and following dexamethasone administration were observed in rats subjected to PPS 58.

Oxidative stress and chronic systemic inflammation constitute important risk factors for cardiovascular diseases such as myocardial infarction or stroke. Similar to PTSD patients 59, an increase in inflammatory cytokines and measures of oxidative stress has recently been demonstrated in rats subjected to PPS 60. There is growing evidence in humans that PTSD increases the risk of cardiovascular events 5. So far, few studies in experimental animals have addressed the effects of psychological trauma on cardio‐ and cerebrovascular vulnerability. Interestingly, a recent study documented increased sensitivity to ischaemic heart injury, that is, larger myocardial infarcts and attenuated post‐ischaemic recovery, in male rats after PPS 61, suggesting that the PPS paradigm may be a useful tool to further explore the association between PTSD and cardiovascular disease.

## Social defeat (SD)

The SD model has been widely used to examine behavioural and physiological sequelae of social stress, mainly in mice 62, 63. SD leads to a long‐lasting increase in social avoidance and submissive behaviour 62. Hammamieh and co‐workers adapted the model to investigate specific aspects of PTSD 64. Mice are exposed to a trained aggressor conspecific for 6 hrs daily for 5 or 10 days in a ‘cage‐within‐cage resident‐intruder’ protocol. It has been shown that animals subjected to SD present persisting PTSD‐like behaviours such as increased freezing, lack of tail rattling 64, enhanced and prolonged response to acoustic startle 65, and weight loss 65. Moreover, SD may produce pathological changes in the heart (inflammatory cardiac histopathologies) and brain (reduced spine density in medial prefrontal cortex) 64.

## Social isolation

Long‐term social isolation (SI) produces relevant behavioural and physiological disturbances in laboratory animals including hyperlocomotion, anxiety‐like behaviour, aggression, cognitive alterations and neuroendocrine changes 66, 67, 68. Mice exposed to 3–4 weeks of SI show increased contextual fear responses and impaired fear extinction 69.

## Early life stress (ELS)

Early life stress (*e.g*. childhood abuse or neglect) significantly increases vulnerability to PTSD in humans 70. ELS is mostly modelled using maternal deprivation. In rats, it has been shown that maternal separation for 3 hrs daily during the first 2 weeks of life leads to an increased acoustic startle reaction, increased anxiety‐like behaviours and hypersecretion of corticosterone in response to mild handling stress in adulthood 71. Moreover, the proportion of rats showing PTSD‐like symptoms in the UT paradigm (see above) increases when the animals have been pre‐exposed to juvenile stress 72.

## Tail suspension

Some models of chronic stress include tail suspension (TS): Mice are suspended by the tail and left dangling in the air for a brief period up to several minutes. This can be combined with an assessment of the animal's behavioural response, which may consist of either struggling/climbing or passive hanging 73. The latter is termed as ‘immobility’ and reflects behavioural despair. Importantly, anti‐depressants may reduce the duration of immobility in the TS procedure 73. Due to the higher body weight of rats, the TS procedure is only used in mice.

## Chronic stress models

So far, chronic stress procedures have primarily been used to model depression 74. However, it appears that chronic stress models may also be studied and interpreted fruitfully in the context of PTSD. Importantly, PTSD criterion A according to DSM‐5 includes repeated traumatization, for example, repeated exposure to accidents in first responders such as firefighters or police officers 26. In the 1980s, Katz and Hersh introduced a 21‐day stress model of anhedonia in the rat 75, which consists of a variety of relatively severe stressors: exposure to 95 dB white noise, repeated unpredictable shock, 40 hrs of food and water deprivation, cold swim at 4.0°C, heat stress at 40°C, shaker stress, reversal of day–night cycle, switch of cage mates and increased housing density. Rats subjected to this chronic stress model show reduced sucrose consumption, a behaviour that is commonly interpreted as a correlate of anhedonia. Katz and co‐workers were also able to demonstrate that anti‐depressants ameliorate the behavioural alterations in their chronic stress model 75.

Willner and colleagues modified the above model slightly, using milder stressors (*e.g*. overnight illumination, shorter periods of food or water deprivation over 4–23 hrs, 30° cage tilt) applied over a longer period of time (5–9 weeks) 76. This so‐called ‘chronic mild stress model’ (CMS) also leads to a decrease in sucrose consumption, disrupted sleep patterns, psychomotor alterations and weight loss, effects that may persist for several months. Again, the behavioural effects of CMS were shown to be reversed by anti‐depressant pharmacotherapy (for review, see 77).

More recently, Strekalova and co‐workers devised a chronic stress model in mice that employs the following stressors over 28 days: exposure to a rat, RS and TS 17. The authors found a strong decrease in sucrose preference in the stressed animals along with, among other things, weight loss, increased anxiety and hyperactivity 17. Interestingly, the development of anhedonia in this stress paradigm was associated with reduced exploration of novelty and increased floating in the forced swim test 17.

Our group adapted this procedure to investigate the effects of chronic stress on mild brain ischaemia 43, 78. We found that mice subjected to chronic stress showed impaired endothelium‐dependent vasorelaxation as well as significantly enlarged brain lesions after 30‐min. middle cerebral artery occlusion (MCAo)/reperfusion 43, 78. The deleterious effects of chronic stress were mediated, at least in part, by increased heart rate because heart rate reduction by ivabradine before stroke induction restored endothelial function and led to a marked reduction of infarct volume 78. HPA system dysregulation in this model was illustrated by the fact that mice subjected to chronic stress showed significantly increased adrenal weights 43. Furthermore, treatment with glucocorticoid receptor (GR) antagonist mifepristone reversed the harmful effects of stress on short‐term stroke outcome at 72 hrs 43.

## Future directions: the role of FKBP5

We are still a long way from understanding why some individuals display a higher vulnerability to develop PTSD after a traumatic event than others. Besides the specific aspects of the traumatic event, other factors such as childhood abuse or neglect, prior experience of trauma, lack of social support and a prior psychiatric history may make an individual more susceptible to PTSD. Genetic factors are also believed to exert an important influence on the risk of PTSD 79, 80. Lately, FK506‐binding protein 51 (FKBP5) has achieved increasing attention as an intracellular mediator of the stress response. Genetic variation and epigenetic modification of FKBP5 have been linked with the development of psychiatric disorders, in particular major depression and PTSD 81, 82, 83, 84. FKBP5 is a co‐chaperone of the GR complex whose reactivity it modulates by decreasing ligand (*i.e*. corticosterone in rodents, cortisol in humans) binding and inhibiting translocation of the receptor complex from the cytosol to the nucleus. Activation of the GR leads to increased FKBP5 transcription, creating an ultra‐fast negative feedback loop 85, 86. The precise pathways through which FKBP5 affects the HPA axis and GR in PTSD and major depression are not fully understood although several recent studies in humans and in experimental animals are beginning to elucidate mechanisms that may underlie the neuroendocrine differences between both disorders.

In survivors of the World Trade Center attacks of 11 September 2001, reduced whole blood messenger RNA (mRNA) expression of FKBP5 has been shown to be associated with PTSD 87, a finding that might lead to enhanced GR responsiveness and explain the HPA axis dysregulation commonly associated with PTSD (*i.e*. lower basal cortisol levels as well as increased feedback sensitivity) 11, 12. Furthermore, exciting findings from a cohort study by Elisabeth Binder and colleagues 81 suggest that polymorphisms of FKBP5 interact with history of childhood abuse to predict onset of PTSD symptoms in adulthood. Interestingly, the authors report that alleles associated with increased induction of FKBP5 confer increased GR resistance in healthy controls, whereas in PTSD patients, these same alleles were associated with increased GR sensitivity 81. This pattern of effects suggests that the relationship between FKBP5 polymorphisms and GR responsiveness is critically dependent on context 88.

Investigations in experimental animals are also beginning to unravel the role of FKBP5 in the neurobiology of depression and PTSD: Along with dysregulation of the HPA axis, SPS has been shown to acutely induce FKBP5 mRNA in prefrontal cortex, hippocampus and amygdala in rats 89. Experiments in FKBP5 knockout mice have shown that these mice are less vulnerable to stress than wild‐type controls 90. Under basal conditions, exploratory drive, locomotor activity, anxiety‐related behaviour, stress‐coping and depression‐like behaviour did not differ between young adult FKBP5^−/−^ mice and littermate controls. However, after different acute stressors, lack of FKBP5 led to a more active coping behaviour 91. Furthermore, FKBP5 deficiency decreased HPA axis reactivity and conferred mild GR hypersensitivity 90, 91. Taken together, these findings suggest that modification of FKBP5 signalling may be a promising strategy for the development of future psychopharmacological agents to treat depression as well as stress‐related disorders.

## Conclusion

PTSD is a devastating psychiatric disorder of high prevalence and major socioeconomic relevance. The biological underpinnings of PTSD are in urgent need of further research. Over the last decades, a rich variety of rodent stress models have been established that faithfully recapitulate certain behavioural and physiological consequences of PTSD. Table 1 summarizes the most relevant experimental procedures, whereas Table 2 provides the comparison of DSM‐5 criteria of PTSD and analogous behaviours in experimental rodents. Input from preclinical neuroscience will be key for further progress on questions related to biological (*e.g*. genetic or epigenetic) determinants of resilience or susceptibility. Of note, genetic models including FKBP5 knockout mice have already provided exciting new insights into the neurobiology of the stress response. Interactions of HPA axis regulation, FKBP5 signalling and the effects of SSRI have been identified in initial studies of chronically stressed animals and remain to be further explored in PTSD 92. Another important area for future experimental research will be to examine how traumatic events and, more broadly, untoward stress conditions, impact the body as a whole 43, 78, 93. Here, interdisciplinary questions pertaining to the interplay between vascular disease (*e.g*. stroke, myocardial infarction) and stress‐related disorders seem particularly pressing.

**Table 1:** Rodent stress models

Model	Method	Behavioural and physiological response
Time‐dependent sensitization (TDS) 27	Different brief stressors (*e.g*. injection of pharmacological agents), followed by re‐stress	Response to subsequent stressor↑, alterations of neurotransmitters, neuroendocrine and immune systems
Foot shock (FS)	Inescapable electric foot shocks, delivered through a steel grid floor, sometimes combined with situational reminder (SR), *e.g*. exposure to shock box	Anxiety behaviour↑, locomotion and rearing ↓, hyperarousal, re‐experiencing 36, freezing response to acoustic triggers ↑ 21, sleep disturbances
Underwater trauma (UT)	Forced swim for 30 sec., followed by submersion for another 30 sec.	Spatial memory ↓ 94
Restraint stress (RS)	Immobilization on wooden boards or in a restraint syringe	Anxiety ↑, nociception ↑
Single prolonged stress (SPS) 42	Restraint for 2 hrs, followed by a forced swim of 20 min. and exposure to ether vapour until unconsciousness. Short session of RS after 7 days	Alterations of HPA axis 42, dysregulation of GABA pathways 48. Freezing in response to contextual fear conditioning ↑, anxiety behaviour ↑, extinction of fear memory ↓ 95
Predator‐based psychosocial stress (PPS)	Exposure to a natural predator or predator‐related stimuli, sometimes combined with instable housing conditions	Prefrontal cortex: serotonin ↓, norepinephrine ↑^96^, inflammatory and oxidative stress markers ↑^60^, anxiety ↑, cognition ↓, startle response ↑, freezing ↑, cognitive skills (*e.g*. memory for new information) ↓ 16
Social defeat (SD)	Exposure to conspecific trained aggressors for 6 hrs daily for 5 or 10 days	Duration of grooming and freezing ↑, locomotion and tail rattling ↓ 64, weight ↓
Social isolation (SI)	Social isolation during 3–4 weeks	HPA system changes, hyperlocomotion, anxiety ↑, aggression, cognitive alterations, freezing ↑, fear extinction ↓ 69
Early life stress (ELS)	Separation of animal pups from their mothers for several hours daily during postnatal days 1–10, followed by re‐exposure to stressor in adulthood	Performance in spatial memory task ↓ 97, contextual freezing and anxiety ↑, startle reflex ↑, neuroendocrine response to subsequent stressors ↑
Tail suspension (TS)	Suspension of mice by the tail	Locomotor activity ↓
Chronic stress model (Katz)	21‐day exposure to severe stressors, *e.g*. loud noise, shock, food and water deprivation, cold swim at 4.0°C, heat stress at 40°C, shaker stress	Sucrose consumption ↓
Chronic unpredictable mild stress (CMS) (Willner)	5–9 weeks exposure to mild stressors, *e.g*. overnight illumination, change of cage mates	Sucrose consumption ↓, disrupted sleep patterns, psychomotor alterations and weight ↓
Chronic mild stress (Strekalova *et al*.) 17	28 days exposure to PPS, RS and TS	Sucrose consumption ↓, vasorelaxation ↓, larger infarct lesions 43

**Table 2:** DSM‐5 criteria and corresponding behavioural phenotypes in animal models

DSM‐5 criteria	Equivalent in animal model
Intrusion	Fear memory: freezing, exaggerated response to mild stressor
Avoidance	Anxiety‐like behaviour in the elevated plus maze
Negative alterations in cognitions and mood	Alteration of spatial memory (*e.g*. Morris water maze task) Reduced social interactions Reduced sucrose consumption Immobility in forced swim test
Alterations in arousal and reactivity	Exaggerated startling response to acoustic stimulus Alterations of sleep Increased locomotor activity Increased aggressive behaviour

## Authors' contributions

All authors participated in the drafting of the final manuscript.

## Ethics approval and consent to participate

Not applicable.

## Conflict of interests

None.

## Funding

This work was supported by the Deutsche Forschungsgemeinschaft (Research grant GE2576/3‐1 to G.K. and K.G.; Exc257 to M.E.), the Bundesministerium für Bildung und Forschung (Center for Stroke Research Berlin to G.K., K.G. and M.E.), the European Union's Seventh Framework Programme (FP7/HEALTH.2013.2.4.2‐1) under grant agreement n° 602354 (Counterstroke consortium to K.G. and M.E.), the German Center for Neurodegenerative Disease (DZNE to M.E.), the German Center for Cardiovascular Research (DZHK to M.E.) and the Corona Foundation (to M.E.).
